# Migration Associated with Climate Change: Modern Face of an Ancient Phenomenon

**DOI:** 10.1289/ehp.120-a205b

**Published:** 2012-05-01

**Authors:** Julia R. Barrett

**Affiliations:** Julia R. Barrett, MS, ELS, a Madison, WI–based science writer and editor, has written for *EHP* since 1996. She is a member of the National Association of Science Writers and the Board of Editors in the Life Sciences.

Climate-related human migration has a long history, with droughts, floods, food shortages, and other climate-related changes forcing the resettlement of populations since early hominids first spread out from Africa nearly 2 million years ago. Human-induced climate change is expected to contribute to even greater population movements in the coming decades, with perhaps as many as 250 million people expected to become “environmental refugees” by 2050. A new review emphasizes that health will be a critical concern regarding climate-related migration [*EHP* 120(5):646–654; McMichael et al.].

Migrations are expected to occur primarily within countries or regions, although some will cross international boundaries; whether they are permanent or short term will depend on the events that prompt them. Extreme weather events such as floods, hurricanes, and heat waves typically lead to short-term internal movement, but slow-onset changes such as land degradation or rising sea levels in coastal areas may force populations to make permanent moves.

As in ages past, migrants seek to survive the consequences of an altered environment. But migration often brings with it new challenges such as food shortages, lack of drinking water, and increased incidence and altered patterns of infectious disease. Developing countries will be particularly affected for two reasons: they lack the infrastructure and resources to cope with climate-related changes, and they often are already contending with pre-existing public health challenges such as malnutrition, lack of medical care, and inadequate infrastructure for water and sanitation.

**Figure:**
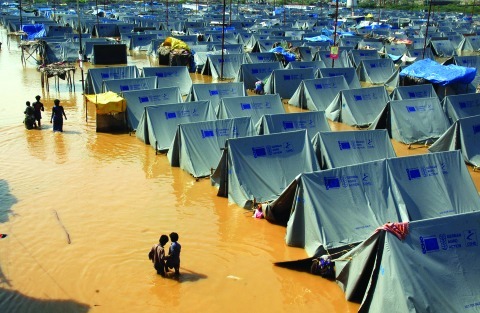
Nearly a year after the 2004 Asian tsunami, displaced Indians still lived in this camp outside Chennai, where they faced fresh environmental health challenges. © Corbis

In this review, migration is classified into three types: forced displacement, planned resettlement, and migration to cities. Each poses a unique but not mutually exclusive set of health risks. For example, populations involved in large-scale forced displacement are at increased risk of infectious disease outbreaks and food shortages. Planned resettlement schemes are typically associated with adverse social outcomes (e.g., land-, job-, and homelessness) that can lead to food insecurity and poor mental health. And poor communities in and around cities—frequently a destination for migrants—are themselves often sited in locations at high risk of climate change impacts, such as low-lying plains and coastal zones. So people migrating into these settings may face continued environmental health threats related not only to poverty but also to climate change.

Migration’s long history means a wealth of experience and knowledge has accumulated in relation to planned resettlement schemes, predisaster planning, and response to humanitarian and environmental disasters. By drawing on this knowledge, as well as undertaking additional research, policy interventions to minimize the adverse health effects of climate change–related migration can be developed. These responses will require coordination, cooperation, and preplanning on multiple levels from local, national, and international agencies and organizations.

